# Sorting by reversals and block-interchanges with various weight assignments

**DOI:** 10.1186/1471-2105-10-398

**Published:** 2009-12-04

**Authors:** Ying Chih Lin, Chun-Yuan Lin, Chunhung Richard Lin

**Affiliations:** 1 Department of Computer Science and Engineering, National Sun Yat-sen University, Kaohsiung 80424, Taiwan; 2 Department of Computer Science and Information Engineering, Chang Gung University, Taoyuan 33302, Taiwan

## Abstract

**Background:**

A classical problem in studying genome rearrangements is understanding the series of rearrangement events involved in transforming one genome into another in accordance with the parsimonious principle when two genomes with the same set of genes differ in gene order. The most studied event is the reversal, but an increasing number of reports have considered reversals along with other genome rearrangement events. Some recent studies have investigated the use of reversals and block-interchanges simultaneously with a weight proportion of 1:2. However, there has been less progress towards exploring additional combinations of weights.

**Results:**

In this paper, we present several approaches to examine genome rearrangement problems by considering reversals and block-interchanges together using various weight assignments. An exact algorithm for the weight proportion of 1:2 is developed, and then, its idea is extended to design approximation algorithms for other weight assignments. The results of our simulations suggest that the performance of our approximation algorithm is superior to its theoretical expectation.

**Conclusion:**

If the weight of reversals is no more than that of block-interchanges, our algorithm provides an acceptable solution for the transformation of two permutations. Nevertheless whether there are more tractable results for studying the two events remains open.

## Background

In comparative genomics, the study of genome rearrangements has been one of the most promising methods for tracing the evolutionary history using gene order comparisons between organisms. The mathematical model simply treats a chromosome in the genome as a permutation of integers, where each integer represents a gene. Specifically, these integers are associated with signs, + or -, to indicate the corresponding orientation (strandedness) of the gene. A basic task in genome rearrangement studies is to economically transform one permutation into another using restricted types of global mutations. Compared with local (point) mutations, global mutations are rare, but can provide valuable clues about the evolutionary history of organisms.

The most widely studied type of global mutations is the *reversal *(also called *inversion*) which inverts a segment in the permutation and changes the sign of each integer in that segment. If we only consider reversals, the so-called problem of *sorting by reversals *(SBR) is to find the shortest series composed of reversals that transforms the given permutation into another, where the minimum number of reversals is often regarded as the (reversal) *distance *between two permutations. SBR is a well-studied subject in computational biology, and its first polynomial-time algorithm was proposed by Hannenhalli and Pevzner in 1995 [1]. Other groups have subsequently simplified and improved this algorithm [2-5]. To date, the best running time of an algorithm for SBR is *O*(*n*^3/2^) in theoretical analysis, as presented by Han [6]. It remains unclear whether SBR can be solved in *O*(*n *log *n*) time, but a plausible answer was recently given by Swenson *et al*. [7], providing two new algorithms; the first runs in randomized *O*(*n *log *n*) time, whereas the other is a deterministic algorithm with running time *O*(*n *log *n *+ *kn*), where *k *is a data-dependent parameter and both its average and standard deviation are small constants derived from extensive experiments [7]. Moreover, a linear-time cost is sufficient to compute the reversal distance [8].

In addition to reversals, *transpositions *and *block-interchanges *are also global mutations that act on a permutation. The former exchanges two adjacent segments, and the latter is a generalization of a transposition in which exchanged segments do not have to be adjacent. The problem of transpositions is called *sorting by transpositions *(SBT), in which the minimum number of transpositions required to complete the transformation is sought. Currently, we know nothing about its complexity, but several approximation algorithms have been proposed [9-11]. However, the problem of *sorting by block-interchanges *(SBBI) using block-interchanges only is tractable and was first studied by Christie [12] using the graph approach and then by Lin *et al*. [13] using the algebraic formalism. Recently, Feng and Zhu [14] introduced a new data structure to improve the approximation and exact algorithms for SBT and SBBI, respectively, to achieve the time complexity *O*(*n *log *n*).

Considering reversals and transpositions together leads to the problem of *sorting by reversals and transpositions *(SBR+T), i.e., it allows one to perform reversals and transpositions alternatively during the transforming process. Because of the two operations used, we assign weights *w*_*r *_to reversals and *w*_*t *_to transpositions, and thus seek a transforming series with a minimum sum of weights. For *w*_*r *_: *w*_*t *_= 1 : 1, Lin and Xue [15] and Walter *et al*. [16] presented approximation algorithms with a factor of 2. By incorporating *inverted transposition*, which inverts one of two swapped segments of a transposition and usually has equal weight *w*_*it *_to *w*_*t*_, in the transformation, 2-approximation algorithms have been reported by two groups [15,17]. Furthermore, Eriksen [18] developed a (1 + *ε*)-approximation algorithm for the weighted assignment of *w*_*r *_: *w*_*t*_(*w*_*it*_) = 1 : 2. Bader and Ohlebusch [19] recently devised a 1.5-approximation algorithm with time *O*(*n*^2^) for any weight proportion of *w*_*r *_: *w*_*t*_(*w*_*it*_) between 1 : 1 and 1 : 2. Nevertheless, it remains unknown whether tractable results can be derived for SBR+T.

In contrast, studying the block-interchanges (with each weight *w*_*bi*_) along with reversals seems easier, i.e., the problem of *sorting by reversals and block-interchanges *(SBR+BI). For *w*_*r *_: *w*_*bi *_= 1 : 2, three groups of researcheres began from different perspectives but all achieved tractable results for SBR+BI [20-22]. Yancopoulos *et al*. [20] introduced a universal *double-cut-and-join *operation that accounts for reversals, *translocations*, *fissions*, *fusions *and block-interchanges by assigning a weight of 2 to block-interchanges and 1 to others. With a slight modification to their algorithm, one can optimally solve SBR+BI [21]. In addition, the approach of Lin *et al*. [21] based on the so-called *breakpoint graph *[1], whereas Mira and Meidanis [22] adopted the algebraic viewpoint by introducing the parameter *norm *to represent the weight of a rearrangement event. By adding a number of local mutations, Bader [23] tackled the problem of unequal gene content using a heuristic algorithm. Despite tractable results when studying SBR+BI under *w*_*r *_: *w*_*bi *_= 1 : 2, to our knowledge, this is the only type of weight assignments that have been considered so far. In this paper, we study genome rearrangement problems by considering reversals and block-interchanges simultaneously using various weight assignments.

On the other hand, a traditional yet effective way to approach a complex problem is to devise an approximate solution that is "not too far from" the exact solution. Approximation algorithms are, indeed, a well-developed branch of the computer sciences [24]. A *β-approximation algorithm *(*β *> 1) for a minimization problem runs in time polynomial to the input size and returns a feasible solution having a quality value that is, at most, *β *times the optimum. More interestingly, since the factor *β *is obtained from the worst-case analysis, an approximation algorithm with a higher factor does not imply poor average performance. To address genome rearrangement problems, two approximation algorithms are developed in this work, together with theoretical analyses and experiments to evaluate their performance.

## Methods

### Preliminaries

A *signed linear permutation * is a permutation of {1, 2, ..., *n*}, where each element is labeled by + or - to indicate the orientation of its corresponding gene. A *reversal r*(*i*, *j*) (with 1 ≤ *i *≤ *j *≤ *n*) is an operation that inverts the order of elements in a segment of  by transforming  into . Another operation, *block-interchange bi*(*i*, *j*, *k*, *l*) (with 1 ≤ *i *≤ *j *<*k *≤ *l *≤ *n*), exchanges two non-intersecting segments () and  by converting  to . For the two operations considered in our study, the weights of reversals and block-interchanges are denoted by *w*_*r *_and *w*_*bi*_, respectively.

Given two permutations  and , the *WGRP*(*w*_*r*_, *w*_*bi*_), abbreviated from *Weighted Genome Rearrangement Problem with w*_*r *_*and w*_*bi*_, is used to find a minimum weighted sequence of reversals and block-interchanges for transforming  into , and its sum of weights  is regarded as the *distance *between  and . In general, the problem is simplified as follows. First, the elements in  and  are relabeled such that  becomes the identity permutation  = (1, 2, ..., *n*), and therefore the transformation from  to  is similar to a sorting process. The distance  is also simplified as *dist*(). Next, for *w*_*r *_> 0, we replace *w*_*bi *_with *w*_*bi*_/*w*_*r *_and fix *w*_*r *_to 1.

When dealing with the signed permutation  of size *n*, most studies extend and transform  into an unsigned mapping *π *= (*π*_0_, *π*_1_, ..., *π*_2*n*+1_) of {0, 1, ..., 2*n *+ 1} beforehand by replacing each positive element *x *of  by 2*x *- 1 and 2*x*, each negative element -*x *by 2*x *and 2*x *- 1, and adding two elements *π*_0 _= 0 and *π*_2*n*+1 _= 2*n *+ 1. For example, if  = (2, -5, -3, -4, -6, 7, 1), then its unsigned mapping is *π *= (0, 3, 4, 10, 9, 6, 5, 8, 7, 12, 11, 13, 14, 1, 2, 15). Each operation on  also corresponds to a specific operation on *π *as follows: A reversal of the form *r*(2*i *+ 1, 2*j*) is said to be *legal *for *π *since it mimics the reversal *r*(*i *+ 1, *j*) on [1], and similarly a block-interchange *bi*(2*i *+ 1, 2*j*, 2*k *+ 1, 2*l*) is *legal *on *π *since it acts like the block-interchange *bi*(*i *+ 1, *j*, *k *+ 1, *l*) on . Considering the above  as an example, the reversal *r*(5, 12) and block-interchange *bi*(1, 8, 11, 14) are legal, whereas *r*(3, 5) and *bi*(1, 9, 11, 14) are not. Furthermore, performing *r*(5, 12) (resp. *bi*(1, 8, 11, 14)) on *π *is equivalent to performing *r*(3, 6) (resp. *bi*(1, 4, 6, 7)) on . In other words, the *WGRP*(*w*_*r*_, *w*_*bi*_) between  and  can be solved by computing a minimum weighted sequence of legal reversals and block-interchanges for converting *π *to *I*. We hereafter use *π *and *I *instead of  and , and legal reversals and block-interchanges in our algorithms.

### Breakpoint graph

**Figure 1:**
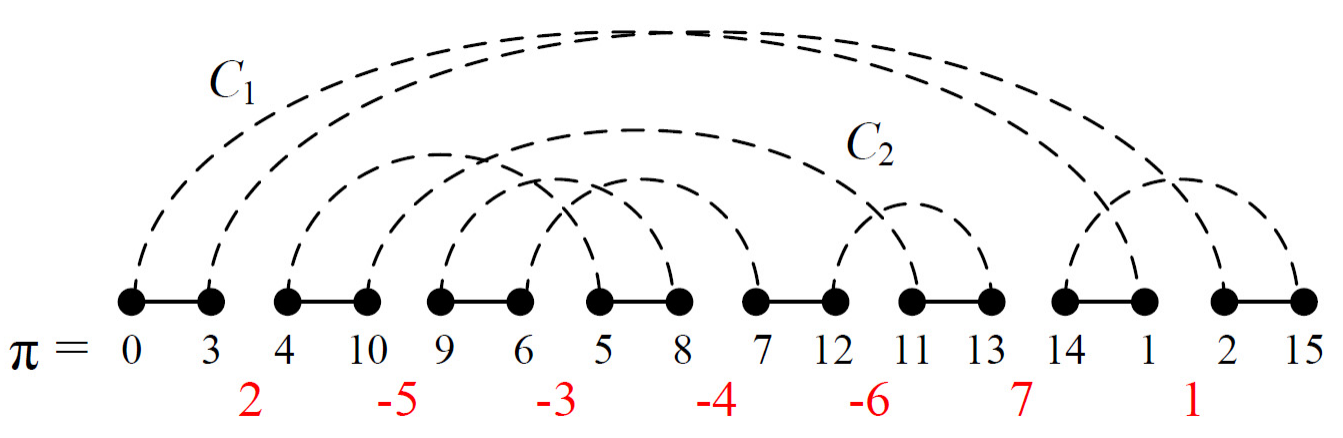
**The breakpoint graph *BP*(*π*) of the permutation *π*, in which black edges are represented as solid lines and gray edges as dashed lines**. The gray edge (4, 5) is oriented whereas (2, 3) is unoriented. In addition, there are two components *C*_1 _and *C*_2_, in which the former is a hurdle.

Let *π *be the permutation mentioned previously. The so-called *breakpoint graph BP*(*π*) is a powerful analysis tool for studying genome rearrangement problems, and is defined as an edge-colored graph with 2*n *+ 2 vertices as follows: For 0 ≤ *i *≤ *n*, *π*_2*i *_connects to *π*_2*i*+1 _by a *black edge *and 2*i *is joined to 2*i *+ 1 by a *gray edge *(Figure 1). In *BP*(*π*), a gray edge (*π*_*i*_, *π*_*j*_) is said to be *oriented *if *i *+ *j *is even, and otherwise it is *unoriented*. A cycle is said to be *alternating *if it contains alternating black and gray edges. Since the degree of each vertex is 2 (a black edge and a gray edge), the graph *BP*(*π*) can be uniquely decomposed into edge-disjoint and alternating cycles. In addition, a cycle is *oriented *as long as it has an oriented gray edge, otherwise, it is *unoriented*. The *length *of a cycle is the number of black (or equivalently, gray) edges it contains. We use *l*-cycle to denote an alternating cycle with length *l*, and *c*(*π*) to denote the number of cycles in *BP*(*π*), e.g., in Figure 1, *c*(*π*) = 2: one is a 5-cycle and the other is a 3-cycle. Note that *c*(*π*) = *n *+ 1 if and only if *π *= *I*.

Each gray edge *g *= (*π*_*i*_, *π*_*j*_) is associated with the interval *< i*, *j *>, and two gray edges *overlap *if their corresponding intervals overlap but neither of them properly contains the other. Moreover, two cycles *overlap *if their gray edges overlap, and a set of overlapping cycles forms a *component*. As with oriented cycles, a component is *oriented *if at least one of its cycles is oriented, and it is *unoriented *otherwise. Using the result of Bader *et al*. [8], the oriented and unoriented components can be efficiently determined in linear time.

A complex and interesting component of the Hannenhalli and Pevzner (HP) theory copes with the *hurdle*, which currently has several slightly different definitions [1,2,18,25,26]. Here we adopt a similar statement to the work of Eriksen [18] but with linear permutations. A *hurdle H *is an unoriented component such that there is an interval containing all vertices in *H *but no vertices in other unoriented components. Here we allow continuous intervals by setting 0 to be the successor of 2*n *+ 1. For the permutation *π *in Figure 1, *C*_1 _is a hurdle since < 12, 15 > ∪ < 0, 1 > is an interval containing the unoriented component *C*_1 _only. Although < 2, 11 > contains *C*_2 _only, *C*_2 _is not a hurdle since it is an oriented component. As a result, the number of hurdles of *π *in Figure 1 is one, i.e., *h*(*π*) = 1.

The HP theory shows that the variations in *c*(*π*) and *h*(*π*) guide the transformation between two permutations. For an arbitrary operation *ρ *acting on *π*, let Δ*c*_*ρ *_= *c*(*ρ*·*π*) - *c*(*π*) and Δ*h*_*ρ *_= *h*(*ρ*·*π*) - *h*(*π*). For convenience, we further abbreviate Δ*c*_*ρ *_(resp. Δ*h*_*ρ*_) to Δ*c*_*r *_(resp. Δ*h*_*r*_) if *ρ *is a reversal and to Δ*c*_*bi *_(resp. Δ*h*_*bi*_) if *ρ *is a block-interchange. HP showed that Δ*c*_*r *_≤ 1 and Δ*h*_*r *_≤ 2 [1]. Christie presented that Δ*c*_*bi *_≤ 2 but on unsigned permutations [12]. A similar argument as Christie's work [12] can extend the upper bound of Δ*c*_*bi *_on signed permutations.

**Lemma 1 ***For every permutation π and block-interchange bi*, Δ*c*_*bi *_≤ 2.

**Figure 2:**
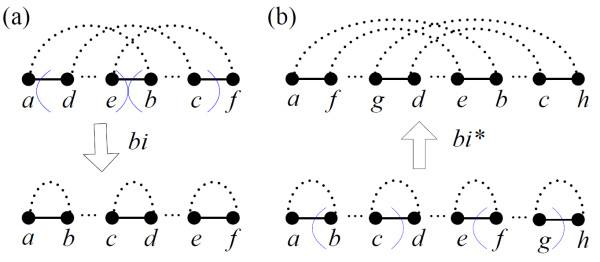
**The block-interchange *bi *defined by (a) three black edges increases the number of cycles by two, whereas (b) four black edges decreases the number of cycles by two**. The pair of blue parentheses specifies one of two exchanged segments of *bi*, and the small dotted lines denote alternating paths.

***Proof***: A block-interchange exchanges two non-overlapping segments, whereas a segment can be specified by two black edges. Let *V*_*bi *_be the set of vertices connected by the black edges for determining the block-interchange *bi*, and *c*(*V*_*bi*_) be the number of cycles containing the vertices in *V*_*bi*_. For example in Figure 2a, *V*_*bi *_= {*a*, *d*, *e*, *b*, *c*, *f*} and *c*(*V*_*bi*_) = 1. According to the number of black edges containing vertices in *V*_*bi*_, we have the following two cases:

*CASE1: Three black edges*. Applying *bi *to *π *affects only the cycles whose vertices are in *V*_*bi*_. Due to the three black edges in this case, we have 1 ≤ *c*(*V*_*bi*_) ≤ 3 and the same is true after applying *bi*, implying that Δ*c*_*bi *_≤ 2 (Figure 2a).

*CASE2: Four black edges*. A similar statement as *CASE1 *shows that Δ*c*_*bi *_≤ 3 as a result of 1 ≤ *c*(*V*_*bi*_) ≤ 4. The only possibility in which Δ*c*_*bi *_= 3 comes from the result of breaking the cycle in *π *into four cycles in *bi*·*π*, but it cannot happen with the subsequent argument. As shown in Figure 2b, the block-interchange bi* with *c*(*V*_bi*_) = 4 results in *c*(*V*_bi*_) = 2 after performing bi*, and hence, Δ*c*_bi* _≠ 1 - 4 = -3. However, if there is a *bi *such that Δ*c*_*bi *_= 3, then the vertices of *V*_*bi *_will be in four cycles of *BP*(*bi*·*π*). Then the bi* exchanging the two swapped segments of *bi *has Δ*c*_bi_* = -3 when it acts on *bi*·*π*, a contradiction. Consequently, Δ*c*_*bi *_≤ 2.   □

#### WGRP(*w*_*r *_= 1, *w*_*bi *_= 2)

For a sorting series *S *= *ρ*_1_, *ρ*_2_, ..., *ρ*_*t *_transforming *π *into *I*, where *ρ*_*i *_represents either a reversal or a block-interchange, let the number of reversals be *d*_*r*_(*S*) and the number of block-interchanges be *d*_*bi*_(*S*). Thus, the weighted sum of *S *is *d*(*S*) = *w*_*r*_·*d*_*r*_(*S*) + *w*_*bi*_·*d*_*bi*_(*S*). The distance *dist*(*π*) is then the minimum *d*(*S*) among all sorting series *S *of converting *π *to *I*. First, we set *w*_*bi *_= 2 and consider *WGRP *(1, 2). Lemma 2 gives a lower bound of *dist*(*π*) in a more general case when 2 ≤ *w*_*bi*_.

**Lemma 2 ***dist*(*π*) ≥ *n *+ 1 - *c*(*π*) *for WGRP*(1, *w*_*bi*_) *with *2 ≤ *w*_*bi*_.

***Proof***: Since Δ*c*_*r *_≤ 1 and Δ*c*_*bi *_≤ 2, an operation increasing the number of cycles by one costs at least , which equals 1 in the case of *w*_*r *_= 1 and 2 ≤ *w*_*bi*_. However, in the best situation, there are at least *n *+ 1 -*c*(*π*) cycles to be increased because of *n *+ 1 cycles in *BP*(*I*). As a result, the cost of any transformation from *π *to *I *is at least *n *+ 1 -*c*(*π*) for *WGRP*(1, *w*_*bi*_) with 2 ≤ *w*_*bi*_.   □

To deal with *WGRP*(1, 2), Lemma 2 shows that if the rearrangement sequences for sorting *π *are composed of reversals with Δ*c*_*r *_= 1 and block-interchanges with Δ*c*_*bi *_= 2, the cost of such a sequence is equal to the lower bound of *dist*(*π*), and hence is optimal. The strategy for selecting best reversals and block-interchanges is the core of the algorithm proposed by Lin *et al*. [21]. Their algorithm distinguished between oriented and unoriented components, and then sorted them separately, i.e., used the algorithm of Kaplan *et al*. [2] to sort all oriented components and the algorithm of Lin *et al*. [13] to deal with the unoriented components. Here we also utilize a known algorithm for SBR, called **ASBR**, to tackle oriented components but we modify the method for sorting unoriented components using the following theorem.

**Theorem 1 ***Let g *= (*π*_*i*_, *π*_*k*_) *and f *= (*π*_*j*_, *π*_*l*_) *be unoriented gray edges of a component. If g and f overlap*, *then there is a block-interchange with *Δ*c*_*bi *_= 2 *in this component*.

**Figure 3:**
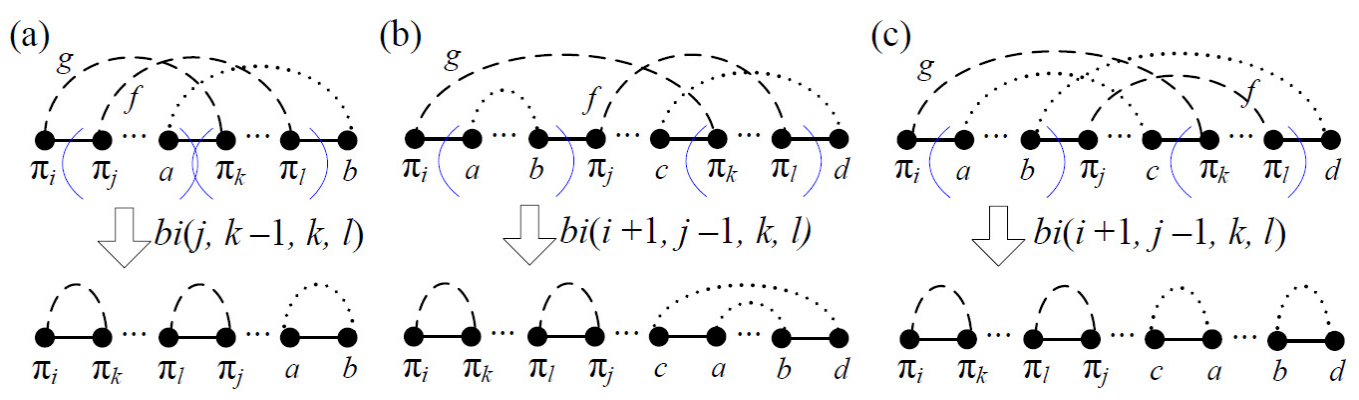
**Two unoriented gray edges *g *= (*π*_*i*_, *π*_*k*_) and *f *= (*π*_*j*_, *π*_*l*_) overlapping in a component are in the same cycle with (a) *j *= *i *+ 1 and (b) *j *>*i *+ 1, whereas (c) *g *and *f *are in different cycles**.

***Proof***: WLOG, we assume that *i *and *l *are even and *j *and *k *are odd with *i *<*j *<*k *<*l *(other cases of *i*, *j*, *k *and *l *can be illustrated similarly). According to the number of cycles containing *g *and *f*, there are two main cases:

*CASE1: g and f are in the same cycle*. We further consider two subcases according to whether *π*_*i *_and *π*_*j *_are connected by a black edge:

(1) *j *= *i *+ 1, i.e., there is a black edge linking *π*_*i *_and *π*_*j *_(Figure 3a). Using the assumption of *k < l*, and that *k *is odd and *l *is even, there is no black edge between *π*_*k *_and *π*_*l*_. Therefore, we use the three black edges, (*π*_*i*_, *π*_*j*_), (*a*, *π*_*k*_), and (*π*_*l*_, *b*) to determine the block-interchange *bi*(*j*, *k *- 1, *k*, *l*). After performing it, the number of cycles is increased by two (Figure 3a), i.e., Δ*c*_*bi *_= 2.

(2) *j *>*i *+ 1. Let *V*_*bi *_= {*π*_*i*_, *a*, *b*, *π*_*j*_, *c*, *π*_*k*_, *π*_*l*_, *d *(Figure 3b). There are no alternating paths from vertex *a *to *c *without passing a vertex in *Vbi*\{*a*, *c*} since *g *and *f *are in the same cycle. Consequently, one of the two cases of alternating paths linking vertices *a, b, c*, and *d *is demonstrated in Figure 3b. In this case, let the block-interchange be *bi*(*i *+ 1, *j *- 1, *k*, *l*) and thus, in *BP*(*bi*(*i *+ 1, *j *-1, *k*, *l*)·*π*) the four vertices, *a*, *b*, *c*, and *d*, belong to one cycle. (The other case can be similarly demonstrated.) We have *c*(*bi*(*i *+ 1, *j *-1, *k*, *l*)·*π*) = *c*(*π*) + 2, which implies that Δ*c*_*bi *_= 2.

*CASE2: g and f are in two different cycles *(Figure 3c). Recall that the order and positions of *i, j, k*, and *l *are fixed via the assumption. On the condition that *g *and *f *are parts of different cycles, *π*_*i *_and *π*_*j *_are never joined by a black edge. In addition, the vertex *a *connects to *b *(or *d*) by an alternating path that will result in the subcase (2) of *CASE1*. As a consequence, Figure 3c is the unique possibility in this case, and performing the block-interchange *bi*(*i *+ 1, *j *- 1, *k*, *l*) leads to Δ*c*_*bi *_= 4 - 2 = 2.   □

All gray edges are unoriented in unoriented components by definition, and furthermore, HP theory presents that for every gray edge *g *not in a 1-cycle, there is another gray edge *f *that overlaps with *g *[1]. In other words, it is always feasible to find two unoriented gray edges overlapping in unoriented components. By repeatedly applying the block-interchanges constructed in Theorem 1, all unoriented components are eventually sorted. We summarize the procedures as **AWGRP(1,2) **as follows:

**Algorithm for *WGRP***(*w*_*r *_= 1, *w*_*bi *_= 2) **(AWGRP(1,2))**

**Input**: A signed permutation .

**Output**: A sorting series composed of reversals and block-interchanges for optimally transforming  into .

**1**: Transform  into its unsigned mapping *π *and construct *BP*(*π*);

**2**: Use the algorithm developed by Bader *et al*. [8] to distinguish between oriented and unoriented components;

**3**: Perform the algorithm of Han [6] to sort all oriented components;

**4**: Repeatedly apply the block-interchanges constructed by Theorem 1 to sort all unoriented components;

**5**: Mimic the sorting series of *π *to *I *to the transformation between  and ;

In **AWGRP(1,2)**, *Step1 *and *Step2 *cost linear time, while *Step5 *can be implemented in *O*(*n *log *n*) time [14,27]. Recently, Feng and Zhu [14] developed a new data structure, called the *permutation tree*, to improve certain algorithms for SBT and SBBI, to achieve the time complexity *O*(*n *log *n*). This group used the permutation tree to implement two core procedures, *Query *and *Transposition*, which were developed by Hartman and Shamir [10] on the breakpoint graph. The former is used to find a pair of black edges *intersecting *the given pair of black edges, and the latter is used to adjust the data structures after applying transpositions. Although the term "intersecting" is defined on black edges [10], it is indeed the same concept as "overlap" here, and thus, can be used to find two overlapping unoriented gray edges to piece together block-interchanges. Moreover, since a block-interchange can be mimicked by two transpositions, a slight modification of the *Transposition *procedure [10] can be applied to retain the structures after performing block-interchanges. In short, the method of Feng and Zhu [14] to enhance the algorithm of Hartman and Shamir [10] can also be extended to cope with performing block-interchanges on unoriented components in *Step4*, for which we do not give a detailed description here. Accordingly, *Step4 *costs *O*(*n *log *n*) time. The running time of *Step3 *is *O*(*n*^3/2^) in a theoretical analysis [6], which is currently the best, or *O*(*n *log *n*) in most cases [7], depending on which algorithm is used to address SBR. As a result, theoretically, the total time complexity of **AWGRP(1,2) **is *O*(*n*^3/2^).

#### WGRP(*w*_*r *_= 1, 2 <*w*_*bi *_< 3)

In this subsection, we adjust the weight of block-interchanges to 2 *< w*_*bi *_< 3 and investigate *WGRP*(1, 2 *< w*_*bi *_< 3). A lower bound of *n *+ 1 *c*(*π*) for *dist*(*π*) is given in Lemma 2, and on the other hand, taking the parameters Δ*h*_*r *_and Δ*h*_*bi *_into account can establish another lower bound. Let Δ(*c-h*)_*r *_= Δ*c*_*r *_- Δ*h*_*r *_and Δ(*c *- *h*)_*bi *_= Δ*c*_*bi *_- Δ*h*_*bi*_. We know that Δ*h*_*r *_≤ 2 and Δ(*c *- *h*)_*r *_≤ 1 from the literature [1], and subsequent work is required to obtain a lower bound of Δ*h*_*bi *_for bounding Δ(*c *- *h*)_*bi*_.

Let *bi *be a block-interchange and *V*_*bi *_be the set of vertices connected to the black edges of *bi*. If a hurdle *H *has no vertices of *V*_*bi *_in its interval ℐ_*H*_, then after performing *bi*, ℐ_*H *_still contains all vertices of *H *but no vertices in other unoriented components, i.e., *H *will be unchanged in *BP*(*bi*·*π*). This provides that Δ*h*_*bi *_≥ -*h*(*V*_*bi*_), where *h*(*V*_*bi*_) is the number of hurdles including vertices of *V*_*bi*_, since there are *h*(*V*_*bi*_) hurdles whose intervals contain the elements in *V*_*bi *_and performing *bi *removes *h*(*V*_*bi*_) hurdles at most. By using the bound for Δ*h*_*bi*_, Lemma 3 immediately derives an upper bound for Δ(*c *- *h*)_*bi*_.

**Lemma 3 ***For every permutation and block-interchange bi*, Δ(*c *- *h*)_*bi *_≤ 3.

***Proof***: Let *c*_*a*_(*V*_*bi*_) be the number of cycles containing vertices of *V*_*bi *_after performing *bi*. Clearly, *c*(*V*_*bi*_), *c*_*a*_(*V*_*bi*_) ∈ {1, 2, 3, 4} and recall that *c*_*a*_(*V*_*bi*_) - *c*(*V*_*bi*_) = *c*(*bi*·*π*) - *c*(*π*) ≤ 2. We prove this lemma by first considering the achievable situations of *c*(*V*_*bi*_) = 4 and *c*_*a*_(*V*_*bi*_) = 4. Lemma 1 demonstrates that the only possibility for *c*_*a*_(*V*_*bi*_) = 4 is Δ*c*_*bi *_= 4 - 2 = 2, in which the two cycles including vertices of *V*_*bi *_belong to a component. Consequently, Δ*h*_*bi *_≥ -*h*(*V*_*bi*_) ≥ -1, and then Δ(*c *- *h*)_*bi *_≤ 2 - (-1) = 3. Using a similar argument, another case of *c*(*V*_*bi*_) = 4 has Δ*c*_*bi *_= 2 - 4 = -2 and *h*(*V*_*bi*_) ≤ *c*(*V*_*bi*_), indicating that Δ(*c *- *h*)_*bi *_≤ -2 - (-4) = 2. Both cases satisfy this lemma.

Next, consider that *c*(*V*_*bi*_), *c*_*a*_(*V*_*bi*_) ∈ {1, 2, 3} is sufficient to show the remaining instances. In these cases, we have Δ*h*_*bi *_≥ -*h*(*V*_*bi*_) ≥ -*c*(*V*_*bi*_), and thus Δ(*c *- *h*)_*bi *_≤ (*c*_*a*_(*V*_*bi*_) - *c*(*V*_*bi*_)) - (*-c*(*V*_*bi*_)) = *c*_*a*_(*V*_*bi*_) ≤ 3. This completes the proof.   □

Next, from Lemma 3, we compute another lower bound for *dist*(*π*). HP proved that one must decrease *dist*_*r*_(*π*) = *n *+ 1 - (*c*(*π*) - *h*(*π*) - *f*(*π*)) to 0 to complete the sorting process if only reversals are allowed, where *f*(*π*) is the characteristic function for the existence of a *fortress*, i.e., *f*(*π*) is 1 if *π *is a fortress and 0 otherwise. In addition, by using a similar argument as Lemma 2, since Δ(*c *- *h*)_*r *_≤ 1 and Δ(*c *- *h*)_*bi *_≤ 3, an operation of increasing *c*(*π*) - *h*(*π*) by one costs at least *min *, which equals  when 2 *< w*_*bi *_< 3. There are, however, at least *n *+ 1 - *c*(*π*) + *h*(*π*) to be increased, leading to a lower bound for *dist*(*π*) in the following lemma.

**Lemma 4 ***dist*(*π*) ≥  (*n *+ 1 *c*(*π*) + *h*(*π*)) *for WGRP*(1, 2 *< w*_*bi *_< 3).

After obtaining two lower bounds of *dist*(*π*), we can evaluate the approximation ratios of two proposed algorithms, **AWGRP(1,2) **and **ASBR**, as they are employed to solve *WGRP*(1, 2 *< w*_*bi *_< 3), where **ASBR **is an algorithm used to optimally solve SBR.

**Theorem 2 ASBR ***is an approximation algorithm for WGRP*(1, 2 *< w*_*bi *_< 3) *with a ratio close to *.

***Proof***: The sorting series given by **ASBR **comprises *dist*_*r*_(*π*) reversals and therefore, to be an approximation algorithm for *WGRP*(1, 2 *< w*_*bi*_< 3), **ASBR **has the factor close to   □

In Theorem 2, we bypass the effect of *f*(*π*) for two reasons: First, the probability that a random signed permutation of size *n *contains a fortress is Θ(*n *^-15^), which is extremely rare [26]. Second, HP illustrated the concept of fortress with a permutation *π *having *dist*_*r*_(*π*) = 23 + 1 - 12 + 3 + 1 = 16 [1], which is, in fact, the minimal *dist*_*r*_(*π*) for a permutation being a fortress. In other words, for *f *(*π*) = 1, the ratio is at most  when 2 <*w*_*bi *_< 3, which is nearly .

**Theorem 3 AWGRP(1,2) ***is a **-approximation algorithm for WGRP*(1, 2 *< w*_*bi *_< 3).

***Proof***: For sorting a permutation *π *with only oriented components, HP presented that *ϕ*(*π*) = *b*(*π*) - *c*(*π*) reversals are sufficient, where *b*(*π*) is the number of black edges in *π*. More specifically, for sorting an oriented component , we need  reversals, in which *b*() (resp. *c*()) is the number of black edges (resp. cycles) in . Similarly, if sorting a set  of oriented components, an **ASBR **will produce  reversals, which is also the same in **AWGRP(1,2)**. When dealing with a set  of unoriented components, **AWGRP(1,2) **constructs  block-interchanges since each decreases  by two.

To convert *π *to *I*, **AWGRP(1,2) **outputs a sorting series with weight sum , and a lower bound of *dist*(*π*) is *ϕ*(*π*) = *n *+ 1 - *c*(*π*) by Lemma 2. As a result, **AWGRP(1,2) **is an approximation algorithm for solving *WGRP*(1, 2 <*w*_*bi *_< 3) with the factor given by   □

Theorems 2 and 3 give the approximation ratios of **ASBR **and **AWGRP(1,2)**, respectively, for approaching *WGRP*(1, 2 <*w*_*bi *_< 3), where their ratios are both at most 1.5. By always selecting the better result of **AWGRP(1,2) **and **ASBR**, we receive a smaller ratio of , whose maximum is /2 ≈ 1.225 when the two terms coincide.

#### WGRP(*w*_*r *_= 1, 1 ≤ *w*_*bi *_< 2)

In the sequel, we readjust the weight of block-interchanges to 1 ≤ *w*_*bi *_< 2 and examine *WGRP*(1, 1 ≤ *w*_*bi *_< 2). Two lower bounds mentioned above,  (*n *+ 1 - *c*(*π*) + *h*(*π*)) and *ϕ*(*π*), are not proper here since the former is too small and the latter is no longer correct. A concise way to obtain a feasible lower bound is to take all oriented components in *π *as unoriented ones. Owing to the increase of at most two cycles by a block-interchange, a lower bound of *dist*(*π*) for *WGRP*(1, 0 <*w*_*bi *_< 2) is .

With the bound, then we have the following theorem.

**Theorem 4 AWGRP(1,2) ***is a **-approximation algorithm for WGRP*(1, 0 <*w*_*bi *_< 2).

***Proof***: Recall that **AWGRP(1,2) **produces a sorting series with *ϕ*() reversals and  block-interchanges. Consequently, to be an approximation algorithm for *WGRP*(1, 0 <*w*_*bi*_< 2), **AWGRP(1,2) **has the factor of   □

Since reversals are main mutations from the evolutionary viewpoint, its weight is often no more than weights of other mutations. Therefore, we focus on improving the algorithm to efficiently cope with *WGRP*(1, 1 ≤ *w*_*bi *_< 2).

**Figure 4:**
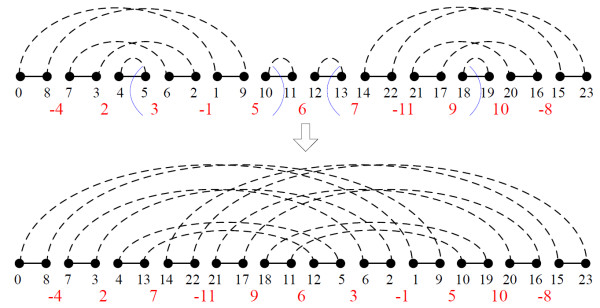
**The illustrated block-interchange merges four oriented components into one at a time**.

We first observed the variation of the approximation ratio in Theorem 4. When *w*_*bi *_is close to 1, the factor  approaches 2, which is insufficient to be used in practice. There are two ways to approach this inefficiency. The first is to make the lower bound higher by considering the fact that block-interchanges do not remove oriented components, and thus, an oriented component has at least one reversal to sort it. However, this does not indicate that  is a new lower bound for *k *oriented components contained in *π*, since an operation may merge most of the oriented components into a single one. Figure 4 is an example of this, and this type of operations may result in the overestimate of  becoming a lower bound. Therefore, we slightly enhance the lower bound by considering that if there is a permutation *π *whose *BP*(*π*) contains an oriented component, then , where the result of *ϕ*(*π*) - 1 is caused by an optimal reversal.

Next, we improve the algorithm by adding a new component. When 1 ≤ *w*_*bi *_< 2, the block-interchange is superior to the reversal since the former decreases *ϕ*(*π*) by at most two whereas the latter decreases it by at most one. Therefore, a straightforward idea is to use optimal block-interchanges whenever possible. Theorem 1 says that if two gray edges are unoriented and overlapping, then the corresponding block-interchange has Δ*c*_*bi *_= 2, which is true regardless of oriented or unoriented components. Nevertheless, there may be no gray edges to satisfy the conditions of Theorem 1 in oriented components. Whenever there are no gray edges to form a block-interchange, we adapt a heuristic method to choose the oriented gray edge *oge *with maximum *P*(*oge*) = *N*(*ooge*) - *N*(*ouge*), where *N*(*ooge*) and *N*(*ouge*) are the number of oriented and unoriented gray edges overlapping with *oge*, respectively.

**Figure 5:**
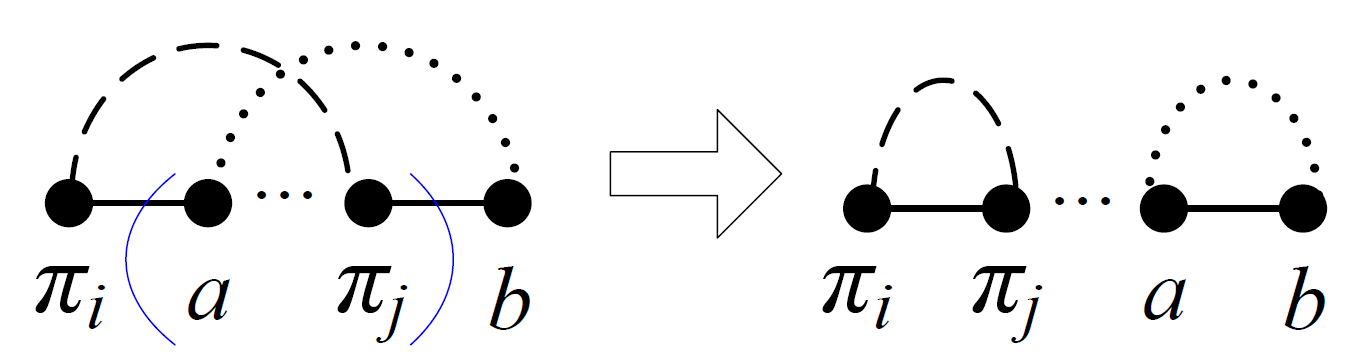
**The reversal specified by a pair of blue parentheses comes from an oriented gray edge (*π*_*i*_, *π*_*j*_), in which *i *and *j *are even**.

Let *oge *= (*π*_*i*_, *π*_*j*_) be an oriented gray edge, and *r*_*oge *_be a reversal defined by two black edges linking *π*_*i *_and *π*_*j*_. Then, we immediately know that *i *+ *j *is even, and hence, both *i *and *j *are either even or odd. The reversal *r*_*oge*_, irrespective of "even" or "odd" case, results in breaking a cycle into two smaller ones, i.e.,  = 1, as demonstrated in Figure 5. Notice that an *oge *can correspond to a reversal having Δ*c*_*r *_= 1, and it is false conversely, i.e., not all optimal reversals can map to oriented gray edges; take  = (-1, -2, -3) and *r*(2, 2) as an example. Besides, a reversal *r*_*oge *_complements the gray edges overlapping with *oge*. In other words, after applying *r*_*oge*_, oriented gray edges overlapping with *oge *become unoriented and vice versa. The heuristic used to compute *P*(*oge*) and select the maximum results from which we want to leave as many unoriented gray edges as possible after performing a reversal. Then, the algorithm is summarized as follows:

**Approximation Algorithm for *WGRP***(*w*_*r*_= 1, 1 ≤ *w*_*bi *_< 2) **(AAWGRP(1,1))**

**Input**: A signed permutation .

**Output**: A sorting series composed of reversals and block-interchanges for transforming  into .

**1**: Transform  into its unsigned mapping *π *and construct *BP*(*π*);

**2: While ***π *is not sorted

**3:**   Repeatedly apply block-interchanges if Theorem 1 holds;

**4:**   Compute *P*(*oge*) for each oriented gray edge *oge*;

**5:**   Select the maximum *P*(*oge*) and perform the corresponding reversal;

6: End while;

**7**: Mimic the sorting series of *π *to *I *to the transformation between  and ;

**Lemma 5 ***After O*(*ϕ*(*π*)) *steps*, *the algorithm ***AAWGRP(1,1) ***stops and returns a sorting series for converting **to *.

***Proof***: Let *π *be the unsigned mapping of . The block-interchanges used in *Step 3 *and reversals in *Step 5 *have Δ*c*_*bi *_= 2 and Δ*c*_*r *_= 1, respectively. In other words, *ϕ*(*π*) = *n *+ 1 - *c*(*π*) is strictly decreased after each applied operation. Due to this fact, **AAWGRP(1,1) **terminates after performing at most *ϕ*(*π*) operations.   □

Now, let us examine the time complexity of **AAWGRP(1,1)**. *Step1 *and *Step7 *are mentioned in **AWGRP(1,2)**, and the two steps require *O*(*n*) and *O*(*n *log *n*) time, respectively. To find two unoriented overlapping gray edges, a linear cost to scan *π *is sufficient. Applying a block-interchange also spends linear time, indicating that the running time to execute *Step3 *once is *O*(*n*). The computation of *P*(*oge*) for an oriented gray edge *oge *can be done simply by visiting the vertices that lay on the interval of *oge *one by one, and then counting the number of oriented and unoriented gray edges overlapping with *oge*, which costs *O*(*n*) time at most. Furthermore, at most *n *computations for *P*(*oge*) implies that *Step4 *can be done within *O*(*n*^2^) time. In *Step5*, an *O*(*n*)-time cost is needed to select the maximum *P*(*oge*) and next perform a corresponding reversal. Therefore, to apply a reversal, the time complexity is *O*(*n*^2^). Finally, **AAWGRP(1,1) **terminates after constructing at most *ϕ*(*π*) operations, and consequently, it takes at most *O*(*n*^3^) time in the worst case.

Comparing **AAWGRP(1,1) **with **AWGRP(1,2)**, the former is preferable to the latter when analyzing oriented components provided that 1 ≤ *w*_*bi *_< 2. **AAWGRP(1,1) **seems feasible for producing a sorting scenario with a smaller sum of weights, but its performance in worst cases is the same as that of **AWGRP(1,2) **for solving *WGRP*(1, 1 ≤ *w*_*bi *_< 2). This is a consequence of certain specific permutations in which their weight sums conducted by both **AAWGRP(1,1) **and **AWGRP(1,2) **are far from the corresponding lower bounds. For example, if *π *has *k *oriented components, each with a 2-cycle only, in its *BP*(*π*), then both **AAWGRP(1,1) **and **AWGRP(1,2) **output *k *reversals; however, the lower bound is just  when *w*_*r *_= *w*_*bi *_= 1. Due to the existence of these challenging cases, the approximation ratio of **AAWGRP(1,1) **is identical to that of **AWGRP(1,2) **when they are used to analyze *WGRP*(1, 1 ≤ *w*_*bi *_< 2).

#### WGRP(*w*_*r *_= 1, 3 ≤ *w*_*bi*_)

*WGRP*(1, 3 ≤ *w*_*bi*_) can be easily solved by considering the fact that an arbitrary block-interchange can be mimicked by three specific reversals. For example, performing the block-interchange *bi*(2, 4, 6, 7) on  = (2, *-*5, -3, -4, -6, 7, 1) is the same as doing three reversals of *r*(2, 5), *r*(3, 7) and *r*(2, 4) in turn on . In other words, as long as a rearrangement sequence consists of a block-interchange, it can be replaced by three corresponding reversals without increasing the weighted sum. As a result, an **ASBR **is sufficient to optimally solve *WGRP*(1, 3 ≤ *w*_*bi*_), and its best running-time to date is *O*(*n*^3/2^) [6].

## Results and Discussion

### Simulation

Despite the appearance of difficult cases with **AAWGRP(1,1)**, it works well in the general situation, even very close to the lower bounds when *w*_*bi *_is near 2. To assess its performance, we conducted several experiments with the sample data generated by applying *αn *operations on  = (1, 2, ..., *n*), where *n *∈ {20, 50, 100} and *α *∈ {0.1, 0.2, ..., 0.9, 1}. The rearrangement operations of either reversals or block-interchanges were selected randomly with equal probability, and each operation was specified at random by selecting two (for reversals) or four (for block-interchanges) integers ranging from 1 to *n*. Moreover, we examined 10*n *test cases and kept track of the mean for each pair of *α *and *n*.

**Figure 6:**
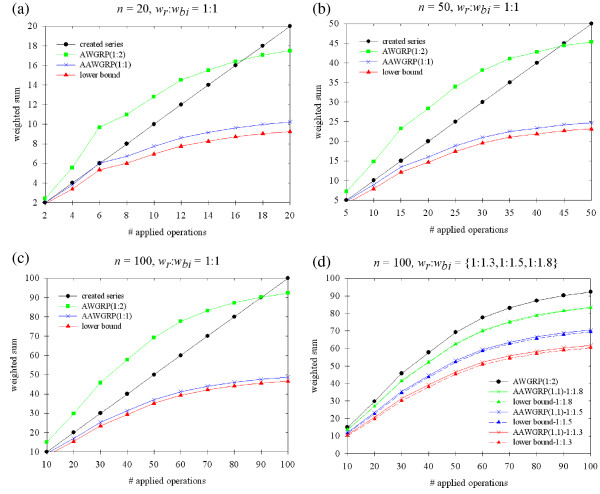
**The diagrams (a), (b) and (c) consist of four curves each whereas (d) has four sets of curves, corresponding to the values of simulations and theoretical estimations**. Specifically in (d), the expression of "**AAWGRP(1,1)**-1:1.3" means that **AAWGRP(1,1) **was used to solve *WGRP*(1, 1.3), and "lower bound-1:1.3" means the lower bound for *WGRP*(1, 1.3).

At the beginning, we considered *WGRP*(1, 1). Then for the simulated data, we computed the corresponding lower bounds as well as the average weight sums of sorting sequences created by **AAWGRP(1,1)**. For comparison, the results of **AWGRP(1,2) **were also marked (Figure 6). The weight sums of four sources, created series, **AWGRP(1,2)**, **AAWGRP(1,1) **and lower bounds, increased with the number of applied operations, but at different rates. Furthermore, in the first three diagrams of Figure 6, regardless of the size *n *or the number of applied operations on permutations, the two curves corresponding to **AAWGRP(1,1) **and the lower bound exhibited the same relative behavior, with only a small gap between them (about 80% of the gaps between the curves were within 2 in the experiment of Figure 6c). This result indicates that **AAWGRP(1,1) **consistently produces a closer estimate of the exact *dist*(*π*) for *WGRP*(1, 1).

Subsequently, in Figure 6d, we fixed *n *= 100 and adjusted *w*_*bi *_= 1.3, 1.5, and 1.8 individually to investigate *WGRP*(1, 1.3), *WGRP*(1, 1.5), and *WGRP*(1, 1.8), respectively. Note that although three problems were included, we only plotted a curve to represent **AWGRP(1,2)**. In addition to simplifying the chart, there was hardly any difference among the reconstructed sequences of **AWGRP(1,2) **for the three problems. In other words, the vast majority of operations in the sorting sequences of **AWGRP(1,2) **were reversals, and hence, their weight sums for the three problems were virtually identical. This phenomenon is expected based on two facts: First, the probability that a component will be unoriented is the same as that of a hurdle, which is Θ(*n*^-2^) on a random permutation of size *n *[26]. Second, the strategy of **AWGRP(1,2) **to remove oriented components is to use an **ASBR **to generate reversals. As a result, the components of the generated permutations are generally oriented, and the sorting sequences of **AWGRP(1,2) **consist mostly of reversals.

Notwithstanding **AWGRP(1,2) **was shown to be a factor 2 approximation algorithm for *WGRP*(1, 1) by Theorem 4, it is indeed infeasible in our experiments. The performance of **AWGRP(1,2) **is gradually improved as *w*_*bi *_moves towards 2 (Figure 6d). In contrast, **AAWGRP(1,1) **improves dramatically when 1 ≤ *w*_*bi *_< 2. Figure 6d suggests that the performance of **AAWGRP(1,1) **is superior to that of **AWGRP(1,2) **in such cases. Even in our simulation of *w*_*bi *_= 1.8, two curves of **AAWGRP(1,1) **and the lower bound were almost the same (most of their differences were less than 1).

### Contribution

A large body of work has been devoted to genome rearrangement problems to study the evolutionary changes in the macrostructure of individual chromosomes according to the parsimonious principle. Here, we investigated the *Weighted Genome Rearrangement Problem *by considering reversals and block-interchanges simultaneously with various weight assignments, i.e., *WGRP*(*w*_*r*_, *w*_*bi*_). Our objective was to find a rearrangement series composed of reversals and block-interchanges for converting  to , as well as the most parsimonious series, that is, the minimum weight sum. We began studying the algorithm *WGRP*(*w*_*r*_, *w*_*bi*_) by setting *w*_*r*_= 1 and *w*_*bi *_= 2, and then developed **AWGRP(1,2) **to optimally solve it. The idea used in **AWGRP(1,2) **is similar to that of Lin *et al*. [21] but differs when coping with unoriented components. We also provided a rigorous proof to show the correctness of **AWGRP(1,2)**.

Furthermore, we adjusted the weight of block-interchanges so that 2 <*w*_*bi *_< 3 to study *WGRP*(1, 2 <*w*_*bi *_< 3). Two algorithms **ASBR **and **AWGRP(1,2) **were employed as approximation algorithms, whose ratios were given by Theorems 2 and 3, respectively. The approximation ratio of **ASBR **is , and hence it decreases if *w*_*bi *_is close to 3; however, the ratio of **AWGRP(1,2) **, which decreases when *w*_*bi *_is near 2. Even if both factors are at most 1.5 for 2 <*w*_*bi *_< 3, their behaviors are completely opposite. Consequently, we obtained a better result by always selecting the best output of the two algorithms to acquire a smaller approximation ratio around 1.225.

Later, the weight of block-interchanges is again varied to fit *WGRP*(1, 1 ≤ *w*_*bi *_< 2). To address this problem, we first showed that **AWGRP(1,2) **is a -approximation algorithm. Nevertheless, the factor becomes larger as *w*_*bi *_moves towards 1. From our experimental results on *WGRP *(1, 1), most of the weighted sums of sorting sequences provided by **AWGRP(1,2) **were more aggravated than the weighted sums of created sequences. Therefore, we improved it with **AAWGRP(1,1) **by adding a new component for selecting operations. Our idea was to choose as many best block-interchanges as possible, and determine plausible candidates for the best reversals once no best block-interchanges were available. As a heuristic, **AAWGRP(1,1) **does not have a smaller approximation ratio than **AWGRP(1,2)**.

Consequently, we conducted several experiments to evaluate its performance and illustrated the results in Figure 6. Our result indicated that, although the theoretical approximation ratio of **AAWGRP(1,1) **trends towards 2 if *w*_*bi *_is close to 1, its average performance is significantly improved. Table 1 further summarizes our current and previous results for solving *WGRP*(*w*_*r*_, *w*_*bi*_).

**Table 1:** Summary of our current and previous results for solving *WGRP*(*w*_*r*_, *w*_*bi*_).

*w*_*r*_	*w*_*bi*_	Results
1	1 ≤ *w*_*bi *_< 2	2/*w*_*bi*_-app. with *O*(*n*^3^) time
	2	*O*(*n*^3/2^)-time algorithm
	2 <*w*_*bi *_< 3	1.225-app. with *O*(*n*^3/2^) time
	3 ≤ *w*_*bi*_	*O*(*n*^3/2^)-time algorithm [6]

## Conclusion

In this work, we present several approaches to examine genome rearrangement problems by considering reversals and block-interchanges together under various weight assignments. Provided that the weight of reversals is no more than that of block-interchanges, our algorithm reports an acceptable solution with theoretical guarantees and experimental evidences. Our results are promising, and these approaches should be used as an initial step for considering the two operations simultaneously. Future research must focus on improving both the approximation ratios and running times of these algorithms.

## Authors' contributions

YCL conceived the research, implemented the program and wrote the manuscript. CYL provided comments and discussion, and also assisted in revising the paper. CRL helped to draft and revise the manuscript. All authors read and approved the final manuscript.
